# *In silico* discovery and validation of potent small-molecule inhibitors targeting the activation function 2 site of human oestrogen receptor α

**DOI:** 10.1186/s13058-015-0529-8

**Published:** 2015-02-25

**Authors:** Kriti Singh, Ravi Shashi Nayana Munuganti, Eric Leblanc, Yu Lun Lin, Euphemia Leung, Nada Lallous, Miriam Butler, Artem Cherkasov, Paul S Rennie

**Affiliations:** Vancouver Prostate Centre, University of British Columbia, 2660 Oak Street, Vancouver, BC V6H 3Z6 Canada; Auckland Cancer Society Research Centre, University of Auckland, Auckland, 1023 New Zealand

## Abstract

**Introduction:**

Current approaches to inhibit oestrogen receptor-alpha (ERα) are focused on targeting its hormone-binding pocket and have limitations. Thus, we propose that inhibitors that bind to a coactivator-binding pocket on ERα, called *activation function* 2 (AF2), might overcome some of these limitations.

**Methods:**

*In silico* virtual screening was used to identify small-molecule ERα AF2 inhibitors. These compounds were screened for inhibition of ERα transcriptional activity using stably transfected T47D-KBluc cell line. A direct physical interaction between the AF2 binders and the ERα protein was measured using biolayer interferometry (BLI) and an ERα coactivator displacement assay. Cell viability was assessed by MTS assay in ERα-positive MCF7 cells, tamoxifen-resistant (TamR) cell lines TamR3 and TamR6, and ERα-negative MDA-MB-453 and HeLa cell lines. In addition, ERα inhibition in TamR cells and the effect of compounds on mRNA and protein expression of oestrogen-dependent genes, pS2, cathepsin D and cell division cycle 2 (CDC2) were determined.

**Results:**

Fifteen inhibitors from two chemical classes, derivatives of pyrazolidine-3,5-dione and carbohydrazide, were identified. In a series of *in vitro* assays, VPC-16230 of the carbohydrazide chemical class emerged as a lead ERα AF2 inhibitor that significantly downregulated ERα transcriptional activity (half-maximal inhibitory concentration = 5.81 μM). By directly binding to the ERα protein, as confirmed by BLI, VPC-16230 effectively displaced coactivator peptides from the AF2 pocket, confirming its site-specific action. VPC-16230 selectively suppressed the growth of ERα-positive breast cancer cells. Furthermore, it significantly inhibited ERα mediated transcription in TamR cells. More importantly, it reduced mRNA and protein levels of pS2, cathepsin D and CDC2, validating its ER-directed activity.

**Conclusion:**

We identified VPC-16230 as an ERα AF2-specific inhibitor that demonstrated promising antiproliferative effects in breast cancer cell lines, including TamR cells. VPC-16230 reduced the expression of ERα-inducible genes, including CDC2, which is involved in cell division. We anticipate that the application of ERα AF2 inhibitors will provide a novel approach that can act as a complementary therapeutic to treat ERα-positive, tamoxifen-resistant and metastatic breast cancers.

**Electronic supplementary material:**

The online version of this article (doi:10.1186/s13058-015-0529-8) contains supplementary material, which is available to authorized users.

## Introduction

Breast cancer (BCa) is the most common type of noncutaneous malignancy and the leading cause of cancer-related death in women worldwide [1,2]. A total of 232,340 new cancer cases and 39,620 cancer deaths were projected to occur in the United States alone in 2013 [3]. Approximately 75% of BCa are classified as oestrogen receptor-alpha (ERα)-positive. Abnormal ERα-mediated activity is the characteristic feature of most of these BCa [4]. The hormone oestradiol (E2) binds to ERα to regulate a wide range of genes associated with proliferation, survival and invasion of breast tumour cells [5,6]. For this reason, the aim of current therapies is to either reduce E2 levels or block signalling through ERα. For the last 30 years, tamoxifen has been the standard treatment for ERα-positive BCa in premenopausal women and for postmenopausal women who have relapsed on aromatase inhibitors. However, most of the patients with advanced disease develop tamoxifen resistance, and one-third of the patients given adjuvant treatment will develop recurrent disease within 15 years of surgery (acquired resistance) due to the progression of the surviving tumour cells to a resistant state [7-9].

Although the factors responsible for development of resistance are not fully understood, several potential mechanisms have been proposed [10]. Altered expression and/or modification of growth factor receptors known to cross-talk with the ERα signalling pathway, such as epidermal growth factor receptor (EGFR), human epidermal growth factor receptor type 2 (HER2) and insulin-like growth factor 1 receptor (IGF-1R) [11-13], and their downstream kinases, such as extracellular signal-regulated kinase 1/2, p38, Akt and p21-activated kinase [14,15], have been shown to correlate with tamoxifen resistance. It is noteworthy that in biopsies from patients with BCa who relapsed on tamoxifen, ERα expression was maintained in more than 50% of cases [16], and up to 80% of metastases from ERα-positive primary tumours retain ERα expression [17,18]. In addition, 20% of patients with resistant disease responded to a second-line treatment of either aromatase inhibitors or fulvestrant [19]. Collectively, these studies suggest the continuing involvement of ERα coregulatory proteins and cross-talk between the ERα pathway and other growth factor and kinase pathways in resistance. Thus, ERα signalling remains an important therapeutic target in resistant disease. Based on these findings, several combination therapies have been proposed [20-22]. However, the problem of resistance remains unresolved, despite the use of the additional endocrine agents. There is a clear unmet need to develop an entirely new class of ERα inhibitor drugs with alternative mechanisms of action to replace or supplement existing treatments.

**Figure 1:**
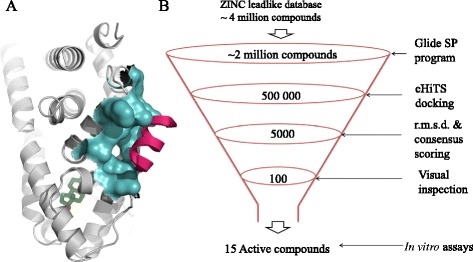
**Graphic representation of oestrogen receptor-alpha activation function 2 pocket and** ***in silico*** **screening pipeline. (A)** Graphic representation of the activation function 2 (AF2) site on the surface of the oestrogen receptor-alpha (ERα) ligand-binding domain (LBD) [PDB:3UUD] AF2 pocket is shown in cyan, coactivator in magenta and oestradiol (E2) in green. **(B)** Virtual screening protocol used for the discovery of AF2 binders. The numbers indicate compounds obtained after each screening step. eHiTS, Electronic high-throughput screening; r.m.s.d., Root mean square deviation.

Targeting alternative sites on the surface of ERα has been proposed as an effective strategy to directly block its activity [23]. The activation function 2 (AF2) site is a coregulator binding site in the ligand-binding domain (LBD) of ERα that recruits a variety of coactivator proteins and mediates diverse functions of ERα. It is a well-characterised, deeply buried hydrophobic cavity that is located topographically adjacent to, but distinct from, the hormone-binding site (HBS) (Figure 1A). Emerging findings suggest that ERα coregulatory proteins are differentially expressed in malignant tumours and that their functions may be altered, leading to tumour progression. For example, coactivators such as steroid receptor coactivator 1 and cAMP-response element-binding protein have been shown to be amplified in BCa [24]. Hao *et al.* suggested that AF2 directly recruits coactivator p300, even in the absence of E2, via the Notch-1 signalling pathway and activates ERα in BCa [25]. Furthermore, Charkavarty *et al.* and Habashy *et al.* independently demonstrated that the ERα AF2 coactivator PELP1 plays a pivotal role in ERα-positive metastasis [26,27]. Collectively, these findings implicate the important role of AF2-mediated functions in ERα-positive BCa. Hence, the coactivator binding site on nuclear receptors has been successfully validated as a bona fide pharmaceutical target in the last decade, and several corresponding drug discovery undertakings have been reported on the subject.

Previously, it has been shown that it is possible to inhibit ERα transcriptional activity by blocking the association of coactivators at the AF2 site. Several groups have identified small-molecule inhibitors of the ERα AF2 site in the past by different approaches [28-37]. However, such compounds have not yet been shown to inhibit the growth of tamoxifen-resistant (TamR) cells. Moreover, the small-molecule inhibitors identified to date demonstrate only moderate micromolar potencies against the ERα and are not yet suitable for clinical application.

In this study, we employed our established computational drug discovery pipeline [38-40] to identify more potent and selective inhibitors of ERα that specifically bind to the AF2 site and inhibit the interaction between ERα and its coactivator proteins. Our lead compound significantly inhibited ERα transcriptional activity by blocking the association of coactivators at the AF2 site. It demonstrated good antiproliferative activity and reduced the expression of ERα-dependent genes. Importantly, it also inhibited the growth of TamR cell lines in an ERα-specific manner.

## Methods

### Preparation of the protein structure for docking

Virtual screening was carried out on the ERα crystal structure [PDB:3UUD] (1.60 Å resolution) [41]. To prepare the protein structure for docking, all solvent molecules were deleted and bond orders for the ligand and the protein were adjusted. The missing hydrogen atoms were added, and side chains were then energy-minimised using the OPLS-2005 force field (as implemented by using Maestro software [42]). The ligand-binding region was defined by a 12 Å box centred on the crystallographic ligand of 3UUD. No van der Waals scaling factors were applied; the default settings were used for all other adjustable parameters.

### Ligand preparation

The ZINC database version 8.0 [43] was used for virtual screening against the ERα AF2 site. The compounds were imported into a molecular database using the Molecular Operating Environment (MOE) version 2012 [44]. Hydrogen atoms were added after these structures were ‘washed’ (a procedure including salt disconnection, removal of minor components, deprotonation of strong acids and protonation of strong bases). The following energy minimisation was performed with the Merck molecular force field 94x, as implemented by the MOE, and optimised structures were exported into the Maestro suite in .sdf format.

### Virtual screening, consensus scoring and voting

Initially, approximately 4 million compounds were docked into the AF2 site using the Glide SP module. Next, we redocked about 2 million molecules, which had a Glide docking score <5.0, into the same binding cavity using the electronic high-throughput screening (eHiTS) docking module. A total of 5 × 10^5^ structures, which received eHiTS docking scores below the 3.0 threshold, were identified for further in *in silico* refinement.

The determined docking poses of the 5 × 10^5^ selected compounds were evaluated by (1) Glide docking score; (2) eHiTS docking score; (3) predicting inhibitory constant (p*K*_i_) of protein-ligand binding using MOE SVL scoring.svl script to improve accuracy of the prediction of energies of hydrogen bonds and hydrophobic interactions; (4) calculating rigorous docking scores, defined by the Ligand Explorer (LigX) module of the MOE package, which accounts for receptor/ligand flexibility; and (5) computing the root mean square deviation (RMSD) between docking poses generated by the Glide and eHiTS programs to quantify their docking consistency. On the basis of these sorted output values from the above four procedures, each molecule would then receive a binary 1.0 vote for every ‘top 10% appearance’. The final cumulative vote (with the maximum possible value of 5) was then used to rank the training set entries. On the basis of the cumulative count, we selected the most highly voted (5 × 10^3^) molecules and subjected their docking poses to visual inspection.

After this final selection step, we formed a list of 100 compounds that were purchased and tested experimentally. Among these 100 compounds, 14 were found to be active and demonstrated the ability to displace the coactivator peptide from the target AF2 site. Small molecules were purchased from established suppliers, including ASINEX (Moscow, Russia; compound VPC-13002), ChemBridge (San Diego, CA, USA; compounds VPC-16007, VPC-16003, VPC-16004, VPC-16222 and VPC-16223), ChemDiv (San Diego, CA, USA; compound VPC-16019), TimTec (Newark, DE, USA; compounds VPC-16041, VPC-16046, VPC-16038, VPC-16040 and VPC-16021) and Vitas-M Laboratory (Apeldoorn, the Netherlands; compounds VPC-16230, VPC-16225, and VPC-16236).

### Cell culture

T47D-KBluc and MDA-MB-453 cell lines were obtained from the American Type Culture Collection (Manassas, VA, USA). The MCF7 and HeLa cell lines were a gift from Dr Sandra Dunn (Division of Hematology and Oncology, Department of Pediatrics, University of British Columbia, Vancouver, BC, Canada). TamR cell lines TamR3 and TamR6 were kindly provided by Dr Euphemia Leung (University of Auckland, New Zealand) [45]. Cells were cultured at 37°C in a humidified incubator with 5% CO_2_. The cell lines were maintained in the following culture media: for MCF7, phenol red–free RPMI 1640 (Gibco/Life Technologies, Grand Island, NY, USA) supplemented with 10% foetal bovine serum (FBS) (Gibco/Life Technologies); for T47D-KBluc, phenol red–free RPMI 1640 containing 4.5 g/L glucose (Sigma-Aldrich, St Louis, MO, USA), 10 mM 4-(2-hydroxyethyl)piperazine-1-ethanesulfonic acid (Sigma-Aldrich), pH 7.5, 1 mM sodium pyruvate (Life Technologies), 0.2 U/ml insulin (Sigma-Aldrich) and 10% FBS; for MDA-MB-453 and HeLa, Dulbecco’s modified Eagle’s medium (HyClone Laboratories/GE Healthcare Life Sciences, Logan, UT, USA) supplemented with 10% FBS (Gibco/Life Technologies); and for TamR3 and TamR6, phenol red–free RPMI 1640 containing 10% charcoal-stripped serum (CSS) (Gibco/Life Technologies) and 1 μM tamoxifen (Sigma-Aldrich).

### Chemicals and antibodies

17β-Oestradiol, 4-hydroxytamoxifen (4-OHT) and tamoxifen were obtained from Sigma-Aldrich. E2 was dissolved in 100% ethanol. 4-OHT, tamoxifen and test compounds were dissolved in dimethyl sulphoxide (DMSO). Cold peroxisome proliferator-activated receptor gamma, coactivator 1-alpha (PGC-1α) peptide (EAEEPSLLKKLLLAPANTQ) was synthesised by Elim Biopharmaceuticals (Hayward, CA, USA).

The rabbit monoclonal anti-pS2 antibody (EPR3972) was obtained from Abcam (Cambridge, UK). The mouse monoclonal antibody against cell division cycle 2 (CDC2) was purchased from (JI-04-00640; RayBiotech, Norcross, GA, USA). The rabbit polyclonal antibody for α-actin (A2066) and mouse monoclonal anti-cathepsin D antibody (C0715) were obtained from Sigma-Aldrich.

### Luciferase oestrogen receptor-alpha transcriptional assay

ERα-positive T47D-KBluc human BCa cells were grown in phenol red–free RPMI 1640 supplemented with 10% CSS for 5 days. The cells were seeded on a 96-well plate (2 × 10^4^ cells/well). After 24 hours, the cells were treated with either the test compounds or 4-OHT in the presence of 1 nM E2. The test compounds were screened at two concentrations: 12 μM and 30 μM. 4-OHT was added at a final concentration of 5 μM. For generation of dose–response curves, the compounds were added at a range of 0.1 to 50 μM and 4-OHT was added at a range of 0.000006 to 3 μM. The medium contained 0.1% (v/v) ethanol and 0.1% (v/v) DMSO. Twenty-four hours after treatment, the medium was aspirated and the cells were lysed by adding 50 μl of 1× passive lysis buffer (Promega, Madison, WI, USA). The plates were placed on a shaker at room temperature for 15 minutes and then subjected to two freeze–thaw cycles to help lyse the cells. Next, 20 μl of the lysate from each treatment was transferred onto a white, 96-well, flat-bottomed plate (Corning Life Sciences, Corning, NY, USA), and the luminescent signal was measured after adding 50 μl of the luciferase assay reagent (Promega) on a TECAN M200 PRO microplate reader (Tecan, Männedorf, Switzerland). Differences in growth were normalised against total protein concentration, which was measured by bicinchoninic acid (BCA) assay.

To rule out binding at HBS, dose–response curves of test compounds (0.1 to 50 μM) and of 4-OHT (0.000095 to 50 μM) were generated in the presence of a set of higher concentrations (1, 10, 50 and 100 nM) of E2 following the same procedure described above.

### Transient transfection

For transient transfection, we used pGL2.TATA.Inr.luc plasmid, which contains three copies of vitellogenin oestrogen response element (ERE) upstream of the TATA promoter (3X ERE TATA luc; Addgene plasmid 11354). This is the same plasmid used to construct pGL2.TATA.Inr.luc.neo, used to create the stable cell line T47D-KBluc.

TamR3 and TamR6 cells were grown in phenol red–free RPMI 1640 supplemented with 10% CSS and 1 μM tamoxifen. The cells were seeded on a 96-well plate (2 × 10^4^ cells/well). After 24 hours, the cells were cotransfected with 50 ng each of the ERα-responsive luciferase plasmid and a constitutive Renilla reporter (to normalise for variations in transfection efficiency) using TransIT-2020 reagent (Mirus Bio, Madison, WI, USA). Cells were treated the next day with the test compounds in the presence of 1 nM E2. The compounds were added in a twofold dilution ranging from 0.1 to 50 μM. Tamoxifen was added at concentrations ranging from 0.000095 to 6 μM, and fulvestrant was added in the range of 0.000095 to 50 μM. The medium contained 0.1% (v/v) ethanol and 0.1% (v/v) DMSO. Twenty-four hours after treatment, the medium was aspirated and the cells were lysed by adding 50 μl of 1× passive lysis buffer (Promega). Luciferase activities were assayed using the Dual-Luciferase Reporter Assay System (Promega).

### Time-resolved fluorescence resonance energy transfer oestrogen receptor-alpha coactivator assay

Peptide displacement was assessed with the LanthaScreen TR-FRET ER Alpha Coactivator Assay kit (PV4544; Life Technologies) as per the instructions of the manufacturer. The compounds were tested in the range of 0.1 to 50 μM, and cold PGC-1α was added at threefold dilutions ranging from 1.8 to 50 μM.

For the peptide competition assay, the compounds were tested in the range of 0.05 to 400 μM in the presence of three different concentrations (250, 500 and 1000 nM) of fluorescein-PGC-1α peptide and the recommended concentrations of glutathione *S*-transferase (GST)-tagged ER-LBD (7.25 nM) and terbium (Tb)-labelled anti-GST antibody (5 nM). Briefly, a twofold serial dilution of the test compounds was prepared at 100× final concentration in DMSO. The compounds were diluted 50-fold in complete assay buffer (assay buffer containing 5 mM dithiothreitol) to get a 2× final concentration and 2% DMSO. The GST-tagged ER-LBD was prepared at 4× final concentration in complete assay buffer, and a 4× fluorescein-PGC-1α/4× Tb anti-GST antibody/4× effective concentration needed to achieve 80% of the maximum response (EC_80_) E2 mix was prepared separately in complete assay buffer. The EC_80_ of E2 was determined to be 6.1 μM in this assay. Ten microlitres of the diluted test compounds were added to a black, flat-bottomed, 384-well plate, followed by addition of 5 μl of the 4× ER-LBD mix. A 4× concentration of fluorescein-PGC-1α/4× Tb anti-GST antibody/4× EC_80_ E2 mix was added last. The plate was incubated at room temperature for 2 hours, and time-resolved fluorescence resonance energy transfer (TR-FRET) was analysed on a Synergy 4 hybrid microplate reader (BioTek, Winooski, VT, USA) with the settings at 340-nm excitation and 495- and 520-nm emission. The emission ratio (520:495) was analysed and plotted.

### Oestradiol displacement assay

E2 displacement was assessed with the PolarScreen ER Alpha Competitor Assay Green kit (P2698; Life Technologies) as per the instructions of the manufacturer. For screening purposes, the compounds were tested at 20 μM in the presence of 25 nM full-length ERα and 4.5 nM fluorescein-labelled E2 (Fl-E2). For E2 ligand competition assay, a twofold serial dilution of the test compounds was prepared at a 100× final concentration in DMSO. The compounds were diluted 50-fold in assay buffer to get a 2× final concentration and 2% DMSO. Fifty microlitres of the diluted test compounds were added to a 50-μl mixture containing 2× full-length ERα and Fl-E2 in each well to obtain final concentrations of 3 to 150 μM of test compound in the presence of 25 nM full-length ERα and 4.5 nM Fl-E2. Unlabelled E2 was tested at concentrations ranging from 0.01 to 1,000 nM. After a 2-hour incubation, polarisation was measured as per the instructions of the manufacturer on a Tecan F500 microplate reader.

### Bio-layer interferometry assay

The direct reversible interaction between small molecules and the ER-α was quantified by BLI using an Octet RED apparatus (Pall ForteBio, Menlo Park, CA, USA). The LDB of the biotinylated oestrogen receptor-alpha (bERα) was produced *in situ* with AviTag technology (Avidity, Aurora, CO, USA). The AviTag sequence (GLNDIFEAQKIEWHE) was incorporated at the N-terminus of the ERα LBD (peptides 302 to 552). A six-residue histidine tag was incorporated at the C-terminus of the ERα LBD for purification of the protein. *Escherichia coli* strain BL21 containing both biotin ligase and ERα LBD vectors was induced with 0.5 mM isopropyl-β-d-1-thiogalactopyranoside in the presence of 0.02 mM E2 and 0.15 mM biotin at 16°C overnight. The bacteria were then lysed by sonication, and the resulting lysate was purified by immobilised metal ion affinity chromatography with nickel agarose beads and cation exchange chromatography (HiTrap SP; GE Healthcare Life Sciences, Pittsburgh, PA, USA). Purified and biotinylated protein (bERα LBD at 0.05 mg/ml) was bound to the Super Streptavidin Sensors (Pall ForteBio) overnight at 4°C in assay buffer (20 mM Tris, pH 7.5, 500 mM NaCl, 0.2 mM tris(2-carboxyethyl)phosphine, 0.02 mM E2, 5% glycerol and 5% DMSO). The compounds were dissolved in the assay buffer in a twofold dilution series ranging from 3.1 to 100 μM. In all experiments, a known AF2-interacting peptide—PGC-1α (Elim Biopharmaceuticals)—was used as a control to confirm the functionality of the bERα LBD.

### MTS assay

Cell proliferation was determined using the 3-(4,5-dimethylthiazol-2-yl)-5-(3-carboxymethoxyphenyl)-2-(4-sulphophenyl)-2*H*-tetrazolium (MTS) assay. Cells were seeded in 96-well plates at a density of 5 × 10^3^ cells/well. MCF7, TamR3, TamR6, MDA-MB-453 and HeLa cells were seeded in their respective media. On the following day, the cells were treated with test compounds (0.2 to 50 μM) in the presence of 1 nM E2 and incubated at 37°C in 5% CO_2_. After 96 hours, 30 μl of MTS reagent (CellTiter 96 AQueous One Solution Cell Proliferation Assay reagent; Promega) were added and incubated for 90 minutes at 37°C in 5% CO_2_. The production of formazan was measured at 490 nm.

### Quantitative RT-PCR

mRNA levels were analysed by quantitative RT-PCR (qRT-PCR). For this purpose, serum-starved MCF7 cells were seeded onto six-well plates at a density of 6 × 10^5^ cells/well. After 24 hours, the cells were treated with the either VPC-16230 or 4-OHT in the presence or absence of 1 nM E2. RNA was isolated after 24 hours with TRIzol reagent (Life Technologies) and purified with the RNeasy Mini Kit (QIAGEN, Valencia, CA, USA). The purified mRNA was quantified using a NanoDrop spectrophotometer (NanoDrop, Wilmington, DE, USA). RNA (0.5 μg) was reverse-transcribed using the iScript synthesis kit (Bio-Rad Laboratories, Hercules, CA, USA). cDNA product (100 ng) was added to the primer mix. The final concentration of the primers was 5 pM. The sequences of the primers used in the qRT-PCR experiments were as follows: pS2, forward 5′-TTGTGGTTTTCCTGGTGTCA-3′ and reverse 5′-GCAGATCCCTGCAGAAGTGT-3′; cathepsin D, forward 5′-CAGAAGCTGGTGGACCAGAAC-3′ and reverse 5′-TGCGGGTGACATTCAGGTAG-3′; CDC2, forward 5′-ACTGGCTGATTTTGGCCTTG-3′ and reverse 5′-TTGAGTAACGAGCTGACCCCA-3′; glyceraldehyde 3-phosphate dehydrogenase (GAPDH), forward 5′-TGCACCACCAACTGCTTAGC-3′ and reverse 5′-GGCATGGACTGTGGTCATGAG-3′. The fold change in expression of the gene was calculated using the 2^−ΔΔ*C*t^ method with GAPDH as the internal control.

### Western blotting

MCF7 cells were serum-starved in phenol red–free RPMI 1640 containing 10% CSS for 5 days. The cells were then seeded onto a six-well plate at a density of 6 × 10^5^ cells/well and treated the following day with the test compounds in the presence of 1 nM E2. After 24 hours, the medium was aspirated, and the cells were washed with ice-cold phosphate-buffered saline. Cells were lysed in 1× radioimmunoprecipitation assay buffer containing one tablet of cOmplete protease inhibitor cocktail (Roche Life Science, Indianapolis, IN, USA). Cell debris was pelleted by centrifugation at 15,000 *g* for 10 minutes at 4°C. The supernatants were collected and quantified using the BCA assay. In each case, 25 μg of protein was loaded onto 15% (v/v) SDS-PAGE gels, separated and transferred to polyvinylidene fluoride membrane. Membranes were incubated with pS2, cathepsin D and CDC2 antibodies or control α-actin antibody. Bound antibodies were detected using horseradish peroxidase–conjugated secondary antibodies. Chemiluminescence was detected with an Amersham ECL detection kit (GE Healthcare Life Sciences), and bands were visualised using the G:BOX imager (Syngene, Frederick, MD, USA).

### Statistical analysis

Data were analysed, and dose–response curves generated, using GraphPad Prism 5 software (GraphPad Software, La Jolla, CA, USA). A *P*-value <0.05 was considered significant.

## Results

In a series of previous studies, we successfully targeted coactivator binding pockets AF2 and binding function 3 (BF3) on the human androgen receptor (AR) and developed several potent AR inhibitors [38-40]. These compounds demonstrated significant antiproliferative effects on a variety of prostate cancer cell lines, including those that are resistant to conventional antiandrogens. Furthermore, several structures of the inhibitors within the intended AR AF2 and BF3 target sites were resolved by X-ray crystallography and deposited in the Protein Data Bank [PDB:4HLW, PDB:2YLO, PDB:2YHD, PDB:3ZQT, PDB:2YLP, PDB:2YLQ]. Because ERα belongs to the same nuclear receptor superfamily, and shares the same domain structural organisation, as the AR, we adopted a similar approach to exploit the ERα AF2 target to develop specific ERα inhibitors. X-ray crystallography studies at the AF2 site have revealed that, despite high sequence homology between the AF2 sites of nuclear receptors, these sites possess different surface shapes and electrostatic characteristics, which may be exploited to achieve selective target binding [23]. These differences allowed us to design inhibitors specific to the ERα AF2 site.

### *In silico* virtual screening for potential activation function 2 binders

The AF2 site represents a hydrophobic groove on the ERα surface flanked by charged amino acids that are essential for the binding of coactivators (Figure 1A). As it is a protein–protein interaction site, AF2 is a challenging target; nevertheless, it offers an attractive option for direct inhibition of ERα activation. Using our in-house computational drug discovery pipeline, we conducted a virtual screen of more than 4 million purchasable compounds from the ZINC database [43] to identify a list of potential AF2 binders. The *in silico* pipeline included large-scale docking, in-site rescoring and consensus voting procedures (for details, see Figure 1B).

We began by collecting and adjusting all chemical structures to their proper protonation state, checking for errors and then docking them into the ERα AF2 pocket using the Glide SP program with default settings [46]. For this purpose, we used the 3UUD crystal structure of the ERα with 1.6 Å resolution [41]. The resulting set of 2 million compounds that received a dock score ≤5.0 were redocked into the 3UUD structure using another docking protocol—eHiTS [47]—with the corresponding docking score threshold set to 3.0. This step helped to reduce a large set of candidate AF2 binders to 5 × 10^5^.

Next, to identify the most consistently predicted binding orientations of the compounds, RMSD was calculated between the docking poses generated by the Glide SP and eHiTS protocols. Only molecules with docking poses with RMSD values <2.0 Å were selected for further analysis. Furthermore, the selected ligands were subjected to additional on-site scoring using the LigX program and the p*K*_i_ prediction module of the MOE [43]. With this information, a cumulative scoring vote of five different parameters (Glide SP score, eHiTS score, LigX score and p*K*_i_ predicted by the MOE) was generated with each molecule, receiving a binary 1.0 score for every ‘top 10% appearance’. The final cumulative vote was then used to select 5,000 compounds that consistently demonstrated high predicted binding affinity toward the targeted AF2 site. These compounds were then visually inspected, and a list of the 100 most promising candidates was determined for purchasing and biological testing.

### Effects of lead compounds on inhibition of transcriptional activity of oestrogen receptor α in breast cancer cell lines

**Figure 2:**
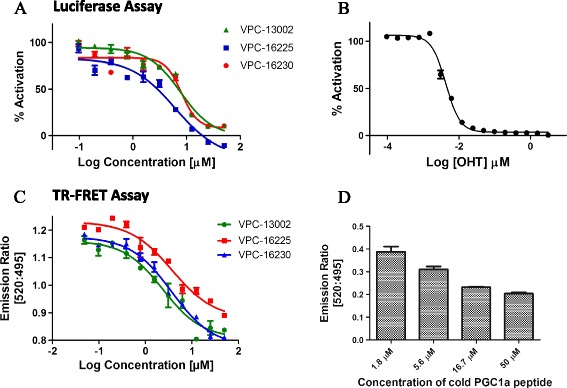
**The lead compounds inhibit oestrogen receptor-alpha transcriptional activity and coactivator binding at the activation function 2 site. (A)** Dose–response curves (0.1 to 50 μM) of compounds VPC-13002, VPC-16225 and VPC-16230 (half-maximal inhibitory concentrations (IC_50_): 7.6, 8.24 and 5.81 μM, respectively) showing inhibition of the oestrogen receptor-alpha (ERα) transcriptional activity as measured by luciferase reporter assay in T47DKBluc cells. **(B)** Dose–response curve (0.000006 to 3 μM) of 4-hydroxytamoxifen (4-OHT) (IC_50_: 4.2 nM) showing inhibition of the ERα transcriptional activity as measured by luciferase reporter assay in T47D KBluc cells. Data were fitted using GraphPad Prism 5 software to calculate the log of the concentration of the inhibitors versus percentage activation. **(C)** Dose–response curves (0.1 to 50 μM) of compounds VPC-13002, VPC-16225 and VPC-16230 (IC_50_: 2.46, 3.76 and 2.98 μM, respectively) for displacement of the PGC-1α peptide from the activation function 2 (AF2) site as measured by time-resolved fluorescence resonance energy transfer (TR-FRET) assay. **(D)** Dose-dependent (1.8 to 50 μM) behaviour of cold peroxisome proliferator-activated receptor gamma, coactivator 1-alpha (PGC-1α) peptide for displacement of the fluorescein-PGC-1α peptide from the AF2 site as measured by TR-FRET assay. Data were fitted using GraphPad Prism 5 software to calculate the log of the concentration of the inhibitors versus the emission ratio. Data points represent the average of two independent experiments performed in triplicates. Error bars indicate standard error of mean for n = 6 values.

The compounds selected from our *in silico* screen were evaluated for their ability to inhibit ERα transcriptional activity using cellular screening assays in ERα-positive T47D-KBluc, a stable luciferase reporter BCa cell line. Twenty active compounds that inhibited the reporter gene expression by at least 50% at 12 and 30 μM were selected for construction of dose–response curves. Among these, 15 compounds demonstrated inhibition of ERα transcriptional activity in a dose-dependent manner, with half-maximal inhibitory concentration (IC_50_) values ranging from 5.8 to 100 μM. Ten compounds summarised in Additional file 1: Table S1 belong to the chemical class of pyrazolidine-3,5–diones, and five compounds from Additional file 2: Table S2 are derivatives of carbohydrazide. Among these, VPC-13002, VPC-16225 and VPC-16230 demonstrated significant inhibition of the reporter gene expression, with IC_50_ values of 7.6, 8.24 and 5.81 μM, respectively (Figure 2A). The IC_50_ of 4-OHT in this assay was established as 4.2 nM (Figure 2B).

### Effect of active compounds on coactivator recruitment to the activation function 2 site

Binding of the identified small molecules to the AF2 site should inhibit E2-dependent coactivator peptide recruitment to this site. To test this hypothesis, the AF2 binders were analysed by using the LanthaScreen TR-FRET ER Alpha Coactivator Assay kit. Tb-labelled anti-GST antibody indirectly labels the ERα LBD by binding to a GST tag on the protein. Binding of the agonist E2 to the ERα causes conformational changes that result in an increase in the affinity of ERα for a fluorescently labelled coactivator peptide, fluorescein-PGC-1α. PGC-1α has been shown to interact with the AF2 site of ERα in an agonist-dependent manner [48]. The close proximity of fluorescein-PGC-1α to the terbium-labelled antibody causes an increase in the TR-FRET signal. When a compound binds to the AF2 site, the recruitment of the coactivator peptide is blocked, causing a decrease in the TR-FRET signal, which is measured as a ratio of emission at 520 nm to 495 nm.

Of 15 chemicals tested, 9 demonstrated effective blocking of AF2–PGC-1α interaction in a concentration-dependent manner, and their IC_50_ values were determined to range between 2 and 20 μM (Additional file 1: Table S1 and Additional file 2: Table S2). This suggests that the compounds bind to the AF2 site, thereby inhibiting coactivator recruitment. The small molecules VPC-13002, VPC-16225 and VPC-16230, which were potent in cellular assays, demonstrated IC_50_ values of 2.46, 3.76 and 2.98 μM, respectively (Figure 2C). The cold PGC-1α peptide used as a control in this assay showed a dose-dependent decrease in the FRET signal with increases in concentration (Figure 2D).

**Figure 3:**
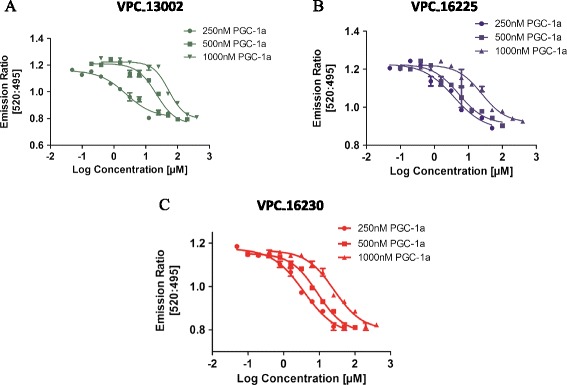
**The dose–response curves of the lead compounds are right-shifted in the presence of higher concentrations of coactivator peptide.** Dose–response curves (0.05 to 400 μM) of **(A)** VPC-13002, **(B)** VPC-16225 and **(C)** VPC-16230 showing a rightward shift with increases in concentrations of fluorescein- peroxisome proliferator-activated receptor gamma, coactivator 1-alpha (PGC-1α) generated by the time-resolved fluorescence resonance energy transfer assay. Error bars indicate standard error of mean for n = 6 values. Data were fitted using GraphPad Prism 5 to calculate the log of the concentration of the inhibitors versus emission ratio.

The three compounds were tested for competition with increasing concentrations of fluorescein-PGC-1α (250, 500 and 1000 nM) to confirm their AF2-mediated mode of action. As expected, a rightward shift in the dose–response curves of the compounds was observed in the presence of higher concentrations of fluorescein-PGC-1α (Figure 3A–C). This suggests that the compounds bind to the AF2 site.

### Active compounds do not bind to the oestrogen receptor-alpha hormone-binding site

**Figure 4:**
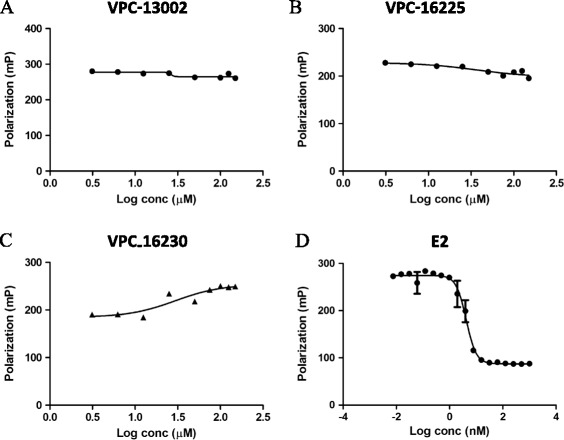
**The lead compounds did not displace oestradiol from the hormone-binding site of oestrogen receptor-alpha.** Dose–response curve (3 to 150 μM) of **(A)** VPC-13002, **(B)** VPC-16225, **(C)** VPC-16230 and **(D)** oestradiol (E2) (0.01 to 1000 nM; half-maximal inhibitory concentration: 4.2 nM) for displacement of fluorescein-labelled E2 in fluorescence polarisation assays. Data were fitted using GraphPad Prism 5 to calculate the log of the concentration of the inhibitors versus polarisation.

For our compounds to be deemed AF2-specific, it was important to confirm that they did not bind to the HBS. To rule out interaction with the HBS, our most active compounds were tested for E2 displacement using the PolarScreen ER Alpha Competitor Assay Green kit. Nine compounds that effectively inhibited coactivator recruitment were tested for E2 displacement at 20 μM. Of the nine compounds tested, six (including VPC-13002, VPC-16225 and VPC-16230) did not exhibit any detectible E2 displacement when tested at 20 μM (see Additional file 3: Figure S1). It should be noted that the *K*_d_ of Fl-E2 with full-length ERα in this assay is reported by the manufacturer as 18 ± 9 nM. Fl-E2 was used at the recommended concentration of 4.5 nM to ensure that the lack of competition observed at 20 μM was not due to the presence of an excess of Fl-E2 ligand. To further confirm that the active compounds did not compete with E2 for binding at the HBS, an E2 ligand competition assay was performed. The results of this experiment demonstrate that these compounds did not displace Fl-E2, even at the highest concentrations tested (3 to 150 μM) (Figure 4A–C), whereas the IC_50_ of unlabelled E2 for displacement of Fl-E2 from the HBS in this assay was 4.2 nM (Figure 4D), which suggests that the estimated cellular IC_50_ values did not reflect binding to the HBS.

**Figure 5:**
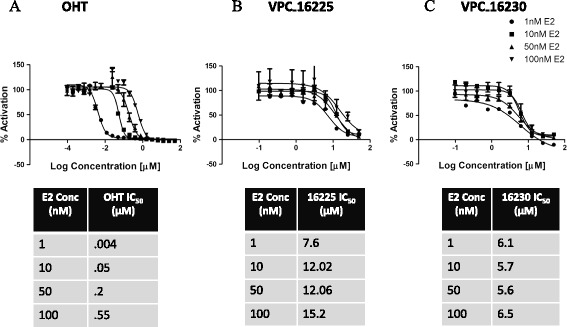
**The dose–response curves of the lead compounds did not shift in the presence of higher concentrations of oestradiol. (A)** Dose–response curves (0.000095 to 50 μM) of 4-hydroxytamoxifen (OHT) showing a rightward shift proportional to the fold increase in concentrations of oestradiol (E2) generated by the luciferase assay in the T47D-KBluc cell line. Dose–response curves (0.1 to 50 μM) of **(B)** VPC-16225 and VPC-16230 **(C)** showing no significant rightward shift in the presence of higher concentrations of E2 as measured by luciferase assay in the T47D-KBluc cell line. Error bars indicate standard error of mean for n = 6 values. Data were fitted using GraphPad Prism 5 to calculate the log of concentration of the inhibitors versus percentage activation.

This was also confirmed by measuring IC_50_ values of the developed inhibitors in the presence of higher concentrations of E2 (luciferase assay with the T47D-KBluc cell line). Because 4-OHT (used as a positive control) competes with E2 for binding at the HBS, we observed a rightward shift in the IC_50_ curve of 4-OHT that was proportional to the fold increase in E2 (Figure 5A). To the contrary, the IC_50_ curves of VPC-16225 and VPC-16230 compounds did not show any significant shift (Figure 5B,C). This confirmed that the compounds did not bind to the HBS.

### Active compounds show direct binding to the oestrogen receptor-alpha ligand-binding domain

To confirm that the identified inhibitors directly bind to the ERα LBD, we cloned and purified the ERα LBD by fusion with an AviTag at the N-terminus and a six-residue histidine tag at the C-terminus. The ERα LBD was biotinylated on the AviTag by a biotin ligase expressed by the bacterial cells cotransformed with the biotin ligase plasmid pBirACm. The bERα LBD was purified by nickel-affinity chromatography and immobilised on streptavidin-coated biosensor tips. The interaction between small molecule and protein was measured in real time as a shift in the interference pattern of the incident light. A response profile was generated on the Octet RED system.

**Figure 6:**
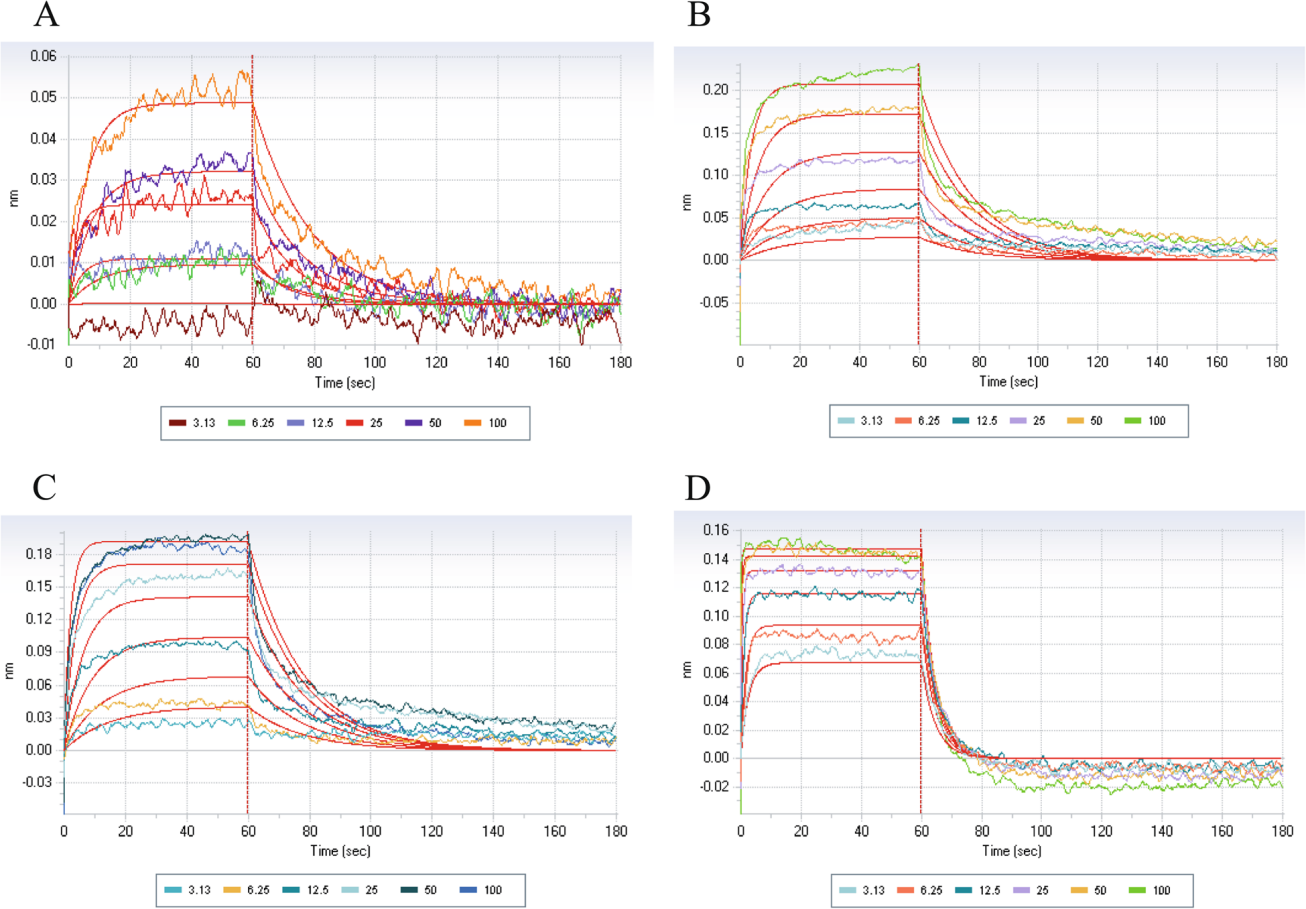
**The lead compounds show direct reversible binding to the oestrogen receptor-alpha ligand-binding domain.** Biolayer interferometry dose–response curves (3 to 100 μM) reflecting the binding of the compounds **(A)** VPC-13002, **(B)** VPC-16225 and **(C)** VPC-16230 to oestrogen receptor-alpha ligand-binding domain in a dose-dependent manner. **(D)** Peroxisome proliferator-activated receptor gamma, coactivator 1-alpha coactivator peptide was used as a positive control.

The binding of the identified six lead compounds was confirmed using this assay. As an example, Figure 6 features the BLI data obtained for the most potent compounds (VPC-13002, VPC-16225 and VPC-16230), along with the PGC-1α peptide used as a control, demonstrating their direct and reversible interaction with ERα. Importantly, it should be noted that the binding curves of these compounds could fit with a simple 1:1 model, even at higher concentrations, suggesting their single-site ER binding.

### Compounds VPC-16225 and VPC-16230 reduce the growth of MCF7 cells, including tamoxifen-resistant cells

**Figure 7:**
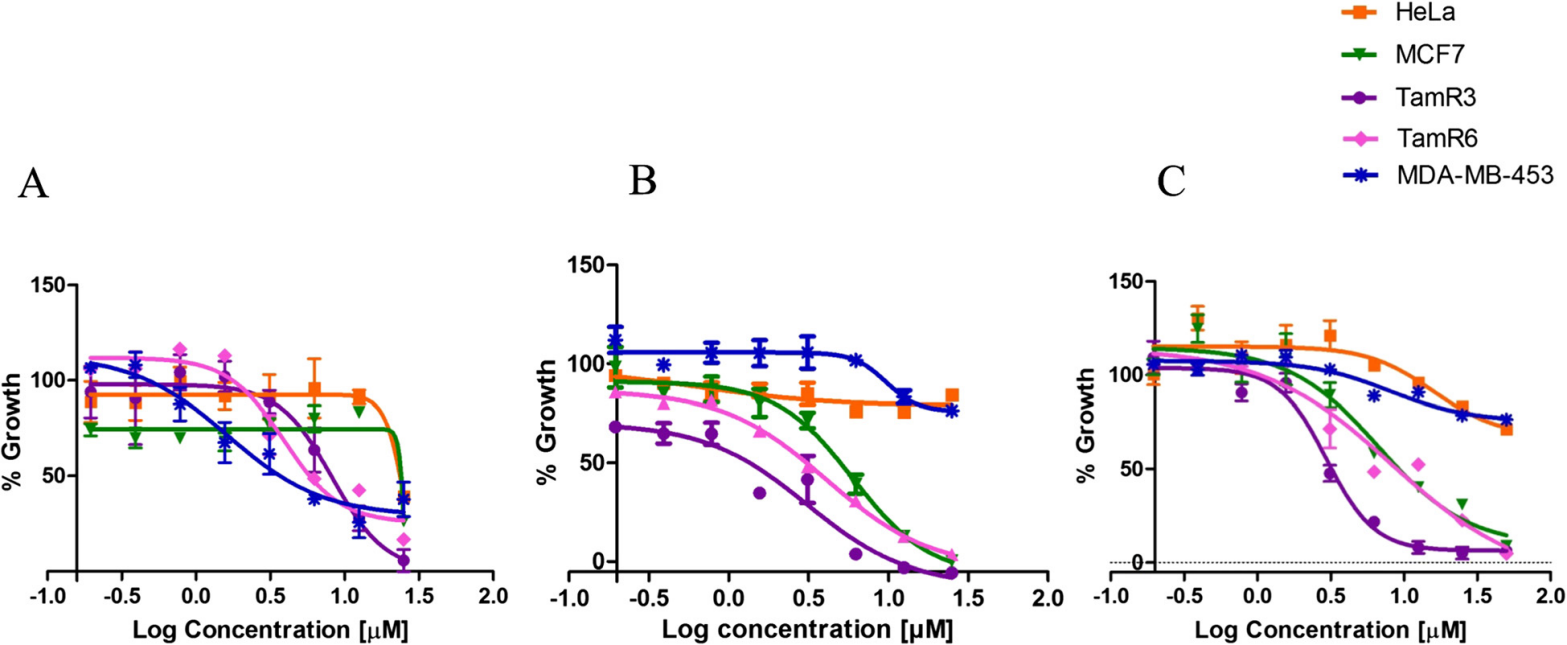
**The lead compounds affect the viability of oestrogen receptor-alpha-positive cells.** Dose–response curves (0.2 to 50 μM) of the lead compounds showing decrease in cell viability as assessed by 3-(4,5-dimethylthiazol-2-yl)-5-(3-carboxymethoxyphenyl)-2-(4-sulphophenyl)-2*H*-tetrazolium assay. The compounds **(A)** VPC-13002, **(B)** VPC-16225 and **(C)** VPC-16230 inhibited the growth of oestrogen receptor-alpha (ERα)-positive MCF7 and tamoxifen-resistant (TamR) cells (TamR3 and TamR6 cell lines) with very little effect on ERα-negative MDA-MB-453 and HeLa cells, except VPC-13002, which was toxic in both the control cell lines. Data points represent average of two independent experiments performed in triplicates. Error bars indicate standard error of mean for n = 6 values. Data were fitted using GraphPad Prism 5 software to calculate the log of concentration of the inhibitors versus percentage growth.

The main objective of the present study was to inhibit the growth of BCa cells by designing small molecules that specifically block the ERα coregulator interaction. To ascertain the growth-inhibitory potential of VPC-13002, VPC-16225 and VPC-16230, we evaluated their ability to inhibit the E2-stimulated growth of ERα-positive, MCF7, TamR3 and TamR6 BCa cells in MTS assays. Cell viability was assessed after 96 hours of incubation with each compound. General cell toxicity was assessed by measuring inhibition of growth in ERα-negative MDA-MB-453 and HeLa cell lines. The VPC-13002 molecule is a derivative of pyrazolidine-3,5-dione that demonstrated certain toxicity in ERα-negative cells; hence, the growth-inhibitory effect of this compound was not considered to be ERα-mediated (Figure 7A), and the molecule was eliminated from further analysis. Figure 7B and C show that carbohydrazide derivatives VPC-16225 and VPC-16230 exhibited growth inhibition of MCF7 cells in a concentration-dependent manner in the range of 0.2 to 50 μM (IC_50_ values of 6 and 7.8 μM, respectively), confirming their ERα-specific effect. Next, we tested VPC-16225 and VPC-16230 in TamR3 and TamR6 cells*.* These cell lines were derived from parental MCF7 cells upon prolonged treatment with tamoxifen and retained expression of ERα. Compared with controls, both VPC-16225 and VPC-16230 inhibited the proliferation of these cell lines in a dose-dependent manner at the concentrations tested. The IC_50_ values for VPC-16225 were 3.1 μM and 4.1 μM in TamR3 and TamR6 cells, respectively. VPC-16230 had IC_50_ values of 3.4 μM and 6.3 μM in TamR3 and TamR6 cells, respectively. It may be noted that, due to the development of resistance, the growth of the TamR3 and TamR6 cell lines was not affected by the presence of 1 μM tamoxifen in the medium.

### Compounds VPC-16225 and VPC-16230 inhibit oestrogen receptor-alpha in tamoxifen-resistant cells

**Figure 8:**
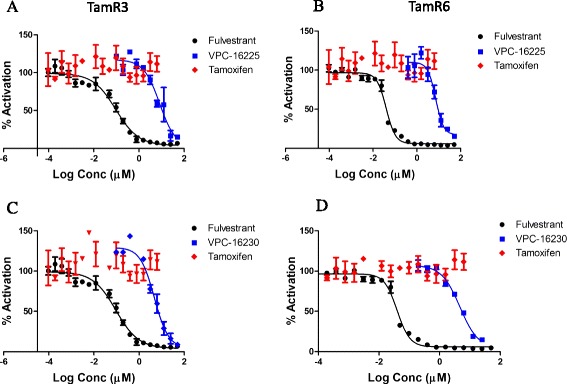
**VPC-16230 and VPC-16225 inhibit oestrogen receptor-alpha transcriptional activity in tamoxifen-resistant cells.** Dose–response curves (0.1 to 50 μM) of compound VPC-16225 for transcriptional inhibition of transiently transfected oestradiol (E2)-responsive luciferase reporter in the tamoxifen-resistant (TamR) cell lines **(A)** TamR3 (half-maximal inhibitory concentration (IC_50_): 8.2 μM) and **(B)** TamR6 (IC_50_: 7.2 μM). VPC-16230 inhibits oestrogen receptor-alpha (ERα) transcriptional activity in a dose-dependent manner in **(C)** TamR3 cells (IC_50_: 4.7 μM) and **(D)** TamR6 cells (IC_50_: 4.7 μM). Dose–response curves (0.000095 to 50 μM) of fulvestrant (IC_50_ in TamR3: 0.09 μM; IC_50_ in TamR6: 0.04 μM) and tamoxifen (0.000095 to 6 μM) are shown for comparison. Data points represent average of two independent experiments performed in triplicates. Error bars indicate standard error of mean for n = 6 values. Data were fitted using GraphPad Prism 5 to calculate the log of the concentration of the inhibitors versus percentage activation.

To confirm that the growth inhibition of TamR cell lines TamR3 and TamR6 was occurring through inhibition of ERα activity, we assessed the ability of VPC-16225 and VPC-16230 to inhibit the expression of an oestrogen-responsive luciferase reporter gene. TamR3 and TamR6 cells were transiently transfected with the luciferase plasmid (3X ERE TATA luc) and then treated the following day with compounds at concentrations ranging from 0.1 to 50 μM, all in the presence of 1 nM E2. Both compounds showed significant inhibition of E2-stimulated ERα transcriptional activity in the two cell lines as measured by the luminescence signal (Figure 8A–D). Fulvestrant, which was used as a positive control in these cells, yielded IC_50_ values of 0.09 and 0.04 μM in TamR3 and TamR6 cells, respectively. As expected, 4-OHT was ineffective in inhibiting the transcriptional activity of ERα in these cells.

### VPC-16230 inhibits expression of oestrogen receptor-alpha-driven genes in MCF7 cells

**Figure 9:**
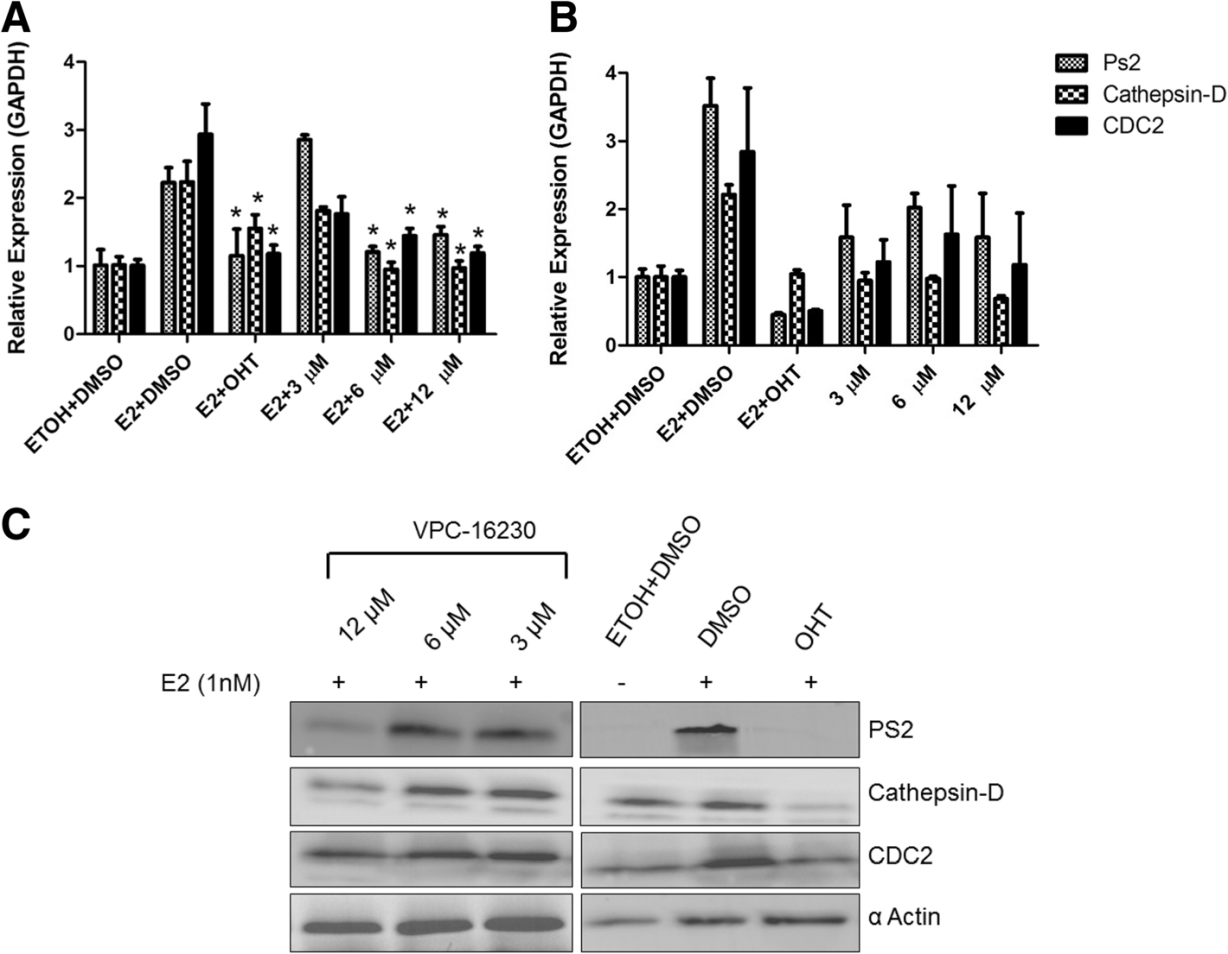
**The lead compounds inhibit mRNA and protein expression levels of oestrogen receptor-alpha-dependent genes. (A)** VPC-16230 significantly decreased mRNA levels of pS2, cathepsin D and cell division cycle 2 (CDC2) at 12 and 6 μM in the presence of 1 nM oestradiol (E2) in MCF7 cells. Data points represent average of two independent experiments performed in triplicates. Error bars indicate standard error of mean for n = 6 values. A *P*-value <0.05 was considered significant (*) compared with E2 + dimethyl sulphoxide (DMSO) control. **(B)** VPC-16230 did not significantly decrease mRNA levels of pS2, cathepsin D and CDC2 at 12, 6 and 3 μM in the absence of 1 nM E2. **(C)** VPC-16230 decreased protein levels of pS2, cathepsin D and CDC2 at 12 and 6 μM in MCF7 cells as observed by Western blot analysis. GAPDH, Glyceraldehyde 3-phosphate dehydrogenase; OHT, 4-Hydroxytamoxifen.

We evaluated the ERα transcriptional inhibitory potential of VPC-16225 and VPC-16230 by measuring the mRNA expression levels of the oestrogen-responsive genes *pS2*, cathepsin D (*CTSD*) and *CDC2* [49,50]. MCF7 cells were treated with the test compounds for 24 hours, following which the mRNA was isolated and qRT-PCR analyses were performed. Whereas VPC-16225 did not show any significant effect, VPC-16230 considerably reduced mRNA levels of these genes in a dose-dependent manner (Figure 9A). However, when treated in the absence of E2, VPC-16230 did not significantly inhibit gene expression compared with the vehicle control (Figure 9B). The inhibition of gene expression in the presence of E2 was also observed at the protein level (Figure 9C). These results suggest that VPC-16230 is a strong inhibitor of ERα transcriptional activity in BCa cells and can be considered as the lead AF2-directed drug prototype in the present study.

## Discussion

Because the AF2 pocket plays a pivotal role in mediating ERα function, targeting this site offers a rich opportunity for the discovery of new BCa drugs. Inhibiting ERα in this manner offers several advantages over conventional anti-oestrogens for the treatment of TamR and metastatic BCa. First, by targeting a site other than the HBS, AF2 inhibitors should be effective in hormone-resistant BCa, because mutations at the HBS should have no effect on the efficacy of AF2 inhibitors. Furthermore, because all conventional anti-oestrogens bind to the same site (that is, the HBS), they cannot be used therapeutically in combinations. By targeting a different site on the ERα, a given AF2 inhibitor could be taken concurrently with the current HBS-directed anti-oestrogen(s). This could potentially be very important for decreasing the time to cancer remission in patients with BCa. Similar to highly active antiretroviral therapy, by taking several complementary therapeutics simultaneously, the rate of drug resistance should be greatly reduced and will lead to an improvement in the overall survival rate of patients with advanced BCa.

### Molecular docking-based analysis of the reported compounds

With the use of *in silico* modelling, we have identified 15 structures that offer two novel classes of molecular scaffolds capable of inhibiting ERα–coactivator interaction at the AF2 site. These *in silico*-identified AF2 binders belong to two distinctive types: derivatives of pyrazolidine-3,5-dione (Additional file 1: Table S1) and carbohydrazide (Additional file 2: Table S2).

**Figure 10:**
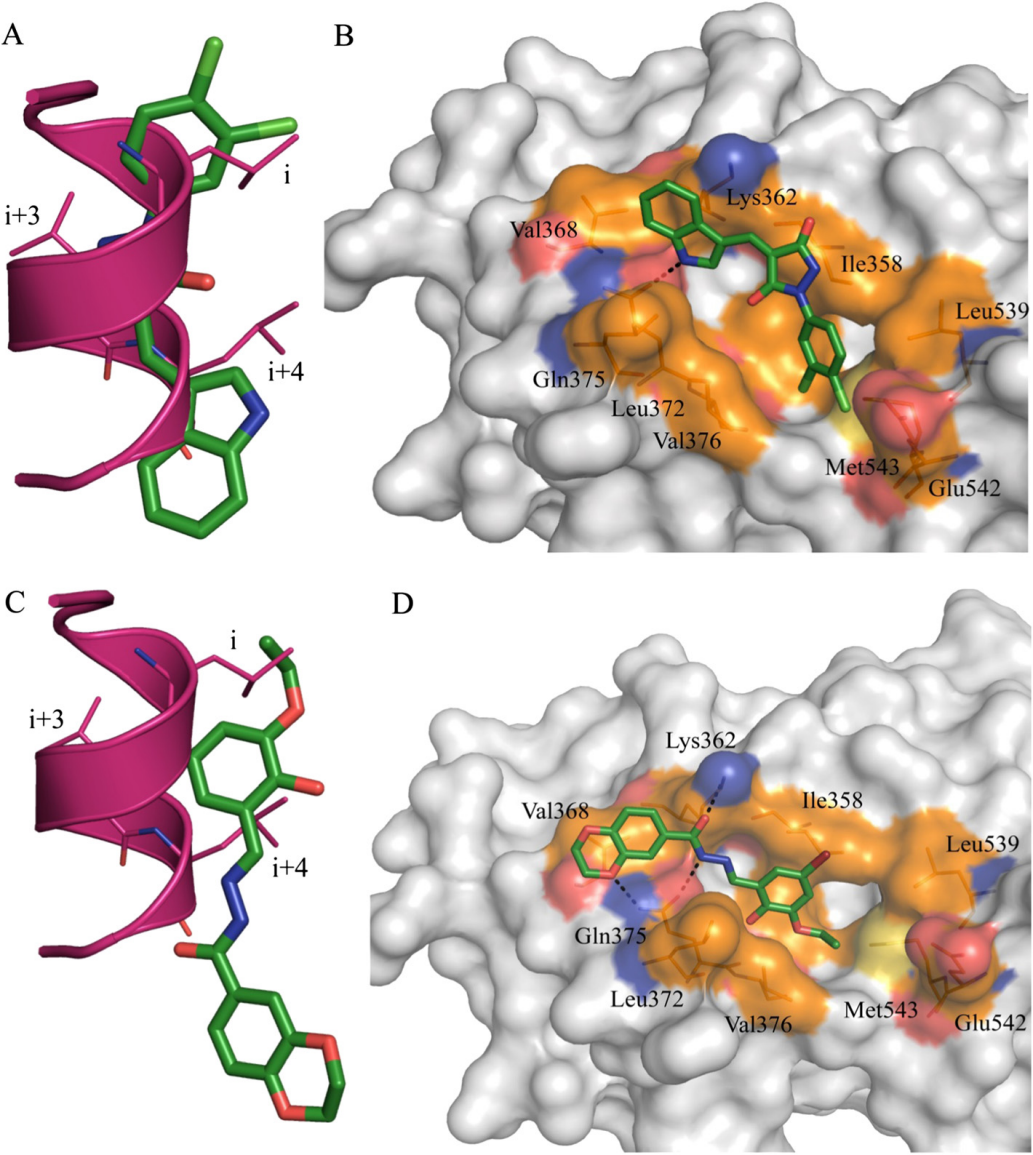
**Binding orientation of VPC-13002 and VPC-16230 inside the oestrogen receptor activation function 2 pocket. (A)** Overlay of the compound VPC-13002 binding pose (green) over the α-helical LXXLL motif (magenta). Indole and aryl groups of VPC-13002 overlap with the leucines at *i* and *i* + 4 positions of the LXXLL motif of the coactivator. **(B)** Predicted binding orientation of VPC-13002 inside the oestrogen receptor (ER) activation factor 2 (AF2) site. **(C)** Overlay of VPC-16230 binding pose (green) over the α-helical LXXLL motif (magenta). **(D)** Predicted binding orientation of VPC-16230 inside the ER AF2 site. Hydrogen bond is shown in black. Hydrophobic residues are shown in brown, positive-charged residues in blue and negative-charged residues in red.

Among our docking models, we found that VPC-13002, a derivative of pyrazolidine-3,5-dione, occupies the AF2 pocket in such a way that it disrupts all key interactions between the ERα AF2 site and the LXXLL motif of the coactivators (Figure 10A). Thus, the indole group of VPC-13002 blocks leucine at the position (*i* + 4) of the motif and forms a strong hydrogen bond interaction with Gln375 (Figure 10B). In addition, Ile358, Lys362, Val368 and Leu372 residues make strong hydrophobic contacts with the indole core. The pyrazolidine moiety of the inhibitor occupies the region of leucine at *i* + 3 and engages with Ile358 residue. Moreover, dichloro moiety of the compound occupies leucine at *i* position and makes additional hydrophobic interactions with Val376, Leu539, Glu542 and Met543.

It should be noted that VPC-16003 and VPC-16004 exhibited a tenfold decrement in peptide displacement and cell inhibition compared with VPC-13002. We can speculate that the replacement of indole- with methyl- or chlorobenzene resulted in losing critical hydrogen bond and hydrophobic interactions. This may account for its lowered peptide displacement activities (IC_50_ > 25 μM versus 2.46 μM for VPC-13002). Because of having the same indole moiety at R1 positions and similar hydrophobic groups at R2 positions, compounds VPC-16019, VPC-16041, VPC-16046 and VPC-16038 demonstrated peptide displacement activities in a comparable range (3.16 to 7.98 μM) with VPC-13002. Compound VPC-16040, with a fourfold benzene substitution, lost its peptide displacement activity fivefold because of the decrease of hydrophobicity at the substitution position. Among all of these compounds, VPC-16021 was the weakest peptide displacer (>100 μM), with poor cell inhibition due to the unavailability of an NH of indole moiety to make a critical hydrogen bond interaction with Gln375. Moreover, lacking hydrophobic groups on the benzene ring hampers its activity. Even though some derivatives of this chemical type demonstrated promising ER-inhibitory activity and effectively block ER coactivator recruitment, they failed to reduce the growth of MCF7 cells. Moreover, VPC-13002 demonstrated toxicity on ER-negative cell lines (Figure 7A). On the basis of the above-described considerations, this class of compounds was not considered in further analysis.

The analysis of the binding orientations of carbohydrazide derivatives, VPC-16230 and VPC-16225 (peptide displacement IC_50_ = 2.98 and 3.76 μM, respectively), inside the target illustrates possible formation of hydrogen bonds with the residues of AF2 ‘charge-clamp’ regions. They perfectly occupy the binding regions of the LXXLL motif and disrupt key interactions between the coactivator and AF2 residues (see Figure 10C for VPC-16230). According to our docking models, VPC-16230 is likely to be anchored by three hydrogen bonds with Lys362 and Gln375. In addition, 5-bromo-3-ethoxy-2-hydroxyphenyl and -benzodioxene moieties of VPC-16230 form strong hydrophobic contacts with Val368, Ile358, Leu372 and V376 (Figure 10D). Notably, bulky substitution on the phenyl ring of VPC-16236 did not allow the compound to form tight binding. Hence, it retained lower peptide displacement activity. It should be noted that the employed peptide displacement assay directly quantifies interactions between the chemicals and the ERα AF2 site and thus provides firsthand evidence of the ER AF2 binding.

### Experimental analysis of the reported compounds

VPC-13002, VPC-16225 and VPC-16230 appeared to be very potent inhibitors of ERα transcriptional activation in cells and AF2 coactivator binding *in vitro*, demonstrating explicit dose–response behaviour (Figure 2A, C). These compounds show a rightward shift in their dose–response curves upon increasing the concentration of the coactivator peptide (Figure 3A–C). This confirms that the compounds are in fact binding at the AF2 site. Further investigation of these molecules using BLI binding assessment, E2 displacement assay and qRT-PCR experiments allowed us to characterise VPC-16230 as the most promising ERα AF2 binder.

In luciferase assays, the IC_50_ of VPC-16230 was established as 5.8 μM (in the presence of 1 nM E2). If we assume that this activity is due to the binding to the HBS part of the receptor, then the binding affinity of the compound is 1/5,800th that of E2. This means that in direct ligand competition assays with Fl-E2, the compound should be able to compete with the fluorescently labelled E2 and its binding affinity should be close to 1/5,800th that of E2. However, when tested in the presence of 4.5 nM Fl-E2, the compound failed to displace the fluorescently labelled ligand, even at concentrations as high as 150 μM (binding affinity approximately 1/30,000th that of E2). The EC_50_ of unlabelled E2 for displacement of Fl-E2 was established at 4.2 nM in this assay. Moreover, if VPC-16230 bound to the HBS, we would observe a shift in its IC_50_ values in the presence of higher concentrations of E2. This was not the case in T47D-KBluc cells, where the inhibition by the compound was tested in the luciferase reporter assay in the presence of 1, 10, 50 and 100 nM E2. The dose–response curve of the compound did not shift significantly with the IC_50_, varying only from 6.1 μM to 6.5 μM with a 100-fold increase in E2 concentration. However, 4-OHT presented a rightward shift of IC_50_ values from 0.004 to 0.5 μM, in proportion to a 100-fold increase in E2 concentration. Taken together, these observations confirm that the mechanism of action of VPC-16230 is not related to HBS binding and is likely to be through AF2 interaction.

Tamoxifen resistance remains a fundamental cause of therapeutic failure in BCa therapy. This creates a challenge for researchers in the field of BCa drug discovery. Studies have shown that the majority of resistant tumours retain dependence on ERα and E2 for survival. In this scenario, AF2-directed compounds could prove critical in suppressing tumour growth. To test this hypothesis, we began by evaluating our compounds in two MCF7-derived TamR cell lines: TamR3 and TamR6. The results of the E2 dose–response experiments with a reporter assay (see Additional file 4: Figure S2), and the effect of fulvestrant on these cells (Figure 8) confirmed that these cell lines retain a functionally active ER. The E2 responsiveness, even in the presence of tamoxifen, suggests that ligand-dependent coactivator recruitment at the AF2 site remains functional in TamR3 and TamR6 cells. Because our lead compounds VPC-16225 and VPC-16230 showed promising inhibition of ER transcriptional activity in an AF2-specific manner, we anticipated that these compounds would also be able to reduce the growth of these resistant cells. When tested in MTS assays, VPC-16225 and VPC-16230 demonstrated promising antiproliferative effects in MCF7, TamR3 and TamR6 cells and had no effect on ER-independent MDA-MB-453 and HeLa cells. This is the first evidence of AF2-directed molecules with an inhibitory effect on TamR cells. Our compounds significantly inhibited the expression of a transiently transfected E2-responsive luciferase reporter in both TamR3 and TamR6 cells, further confirming that the reduction in the growth of TamR3 and TamR6 is via ER inhibition through AF2-directed action.

A comparison of the binding modes of AF2 ligands and tamoxifen (Additional file 5: Figure S3) revealed distinct differences in the structural conformation of ER. Tamoxifen allosterically affects coactivator recruitment by competitively inhibiting association between E2 and ER HBS. This causes conformational changes in the receptor that eventually lead to movement of helix 12 in such a way that it prevents the formation of the AF2 site. Because E2 continues to activate ERα, the AF2 site is still accessible and the compounds can bind the pocket and inhibit the receptor in TamR3 and TamR6 cells. This provides a possible explanation why AF2 inhibitors are effective in these cells, as observed in our assays.

The downstream effect of ER inhibition should be reflected as downregulation of ER target genes. VPC-16230 could significantly decrease E2 stimulated mRNA expression levels of ERα target genes such as pS2, cathepsin D and CDC2 in MCF7 cells. However, when treated in the absence of E2, the compound did not show any significant effect on the expression levels of these genes. This was expected because E2 is required for the AF2 pocket to be formed and because, in its absence, the compounds will not bind to the AF2 site and therefore will not show any effect. It is well established that E2 stimulates the proliferation of MCF7 cells, and it was observed that CDC2, a cell cycle–associated gene whose expression was increased threefold upon E2 stimulation, was significantly reduced by VPC-16230. This observation correlates with the inhibition of proliferation by VPC-16230 of MCF7 cells in MTS assays. Because these genes are ERα-dependent, their inhibition corroborates the idea that blocking coactivator binding is a promising approach to inhibit ER activity in BCa cells.

Overall, our results indicate that targeting the AF2 site with small molecules represents a feasible therapeutic approach that allows successful inhibition of growth of TamR BCa cells. Although the potency of our lead AF2 inhibitor, VPC-16230, is less than that of tamoxifen, one must note that this is an early-stage compound and that its activity can be enhanced by further lead optimisation efforts.

## Conclusions

In BCa, progression to a hormone-resistant phenotype in the presence of an ERα antagonist is possibly maintained through selection of cells with epigenetic or mutational changes that bypass the inhibitory action of the drug [48,51,52]. Using a combination of virtual screening and biochemical assays, we have identified several compounds that bind to the AF2 site of human ERα and directly inhibit its interaction with coactivators. The present study is the first successful attempt to identify direct disruptors of ERα transactivation using an *in silico* virtual screening approach, and this resulted in the identification of 15 AF2-selective low micromolar inhibitors. On the basis of these results, more potent ERα inhibitors should be investigated, with the goal of developing novel therapeutic strategies that can act as complementary approaches to treat ERα-positive, TamR and metastatic BCa.

## Additional files

Additional file 1: Table S1. Structures and measured activities of the identified ER AF2 inhibitors (pyrazolidine-3,5-dione derivatives). Additional file 2: Table S2. Structures and measured activities of the identified ER AF2 inhibitors (acetohydrazide derivatives). Additional file 3: Figure S1. E2 displacement of VPC-13002, VPC-16225 and VPC-16230. The lead compounds were tested at 20 μM for E2 displacement in fluorescence polarization assay. The compounds did not displace Fl-E2. OHT (0.5 μM) and E2 (2 μM) were used as positive controls. Additional file 4: Figure S2. E2 dose–response profile of MCF7, TamR3 and TamR6. Dose response to E2 was measured in the three cell lines by luciferase reporter assay using the E2-responsive 3X ERE TATA luc plasmid. **(A)** In the presence of 1 μM tamoxifen, both MCF7 and TamR cell lines show a dose-dependent response to E2; however, in MCF7, the effect is diminished at lower concentrations of E2. **(B)** In the absence of 1 μM tamoxifen, MCF7 shows a dose-dependent behaviour with a significantly higher luminescent signal, whereas in TamR cell lines the response is similar to that in the presence of 1 μM tamoxifen. Additional file 5: Figure S3. Antagonist and agonist models of estrogen receptor-α. **(A)** Binding of tamoxifen (shown in purple) to ER-α leads to an antagonist conformation, which results in repositioning of αα-helix 12 (show in red) and prevents AF2 formation. **(B)** Binding of E2 (shown in cyan) causes the movement of α-helix 12 such that it opens the AF2 pocket. In this scenario, AF2 inhibitor (shown in green) directly prevents interaction between coactivators and the receptor.

## Abbreviations

AF2: Activation function-2
AR: Androgen receptor
BCa: Breast cancer
BCA: Bicinchoninic acid
bERα: Biotinylated oestrogen receptor-alpha
BF3: Binding function 3
BLI: Biolayer interferometry
CDC2: Cell division cycle 2
CSS: Charcoal-stripped serum
DMSO: Dimethyl sulphoxide
E2: Oestradiol
EC_80_: Effective concentration needed to achieve 80% of the maximum response
EGFR: Epidermal growth factor receptor
eHiTS: Electronic high-throughput screening
ERα: Oestrogen receptor-alpha
ERE: Oestrogen response element
FBS: Foetal bovine serum
Fl-E2: Fluorescein-labelled oestradiol
GAPDH: Glyceraldehyde 3-phosphate dehydrogenase
GST: Glutathione *S*-transferase
HBS: Hormone-binding site
HER2: Human epidermal growth factor receptor type 2
IC_50_: Half-maximal inhibitory concentration
IGF-1R: Insulin-like growth factor 1 receptor
LBD: Ligand-binding domain
LigX: Ligand Explorer module
MOE: Molecular Operating Environment
MTS: 3-(4,5-dimethylthiazol-2-yl)-5-(3-carboxymethoxyphenyl)-2-(4-sulphophenyl)-2*H*-tetrazolium
4-OHT: 4-Hydroxytamoxifen
PGC-1α: Peroxisome proliferator-activated receptor gamma, coactivator 1-alpha
qRT-PCR: Quantitative real-time reverse transcriptase polymerase chain reaction
RIPA: Radioimmunoprecipitation assay
TamR: Tamoxifen-resistant
TR-FRET: Time-resolved fluorescence resonance energy transfer
