# Supplementary figures and images for: In silico discovery and validation of potent small-molecule inhibitors targeting the activation function 2 site of human oestrogen receptor α
Source: Breast Cancer Res. 2015 Feb 25;17(1):27. doi: 10.1186/s13058-015-0529-8 (PMC4360945; doi:10.1186/s13058-015-0529-8)

## Slide 1
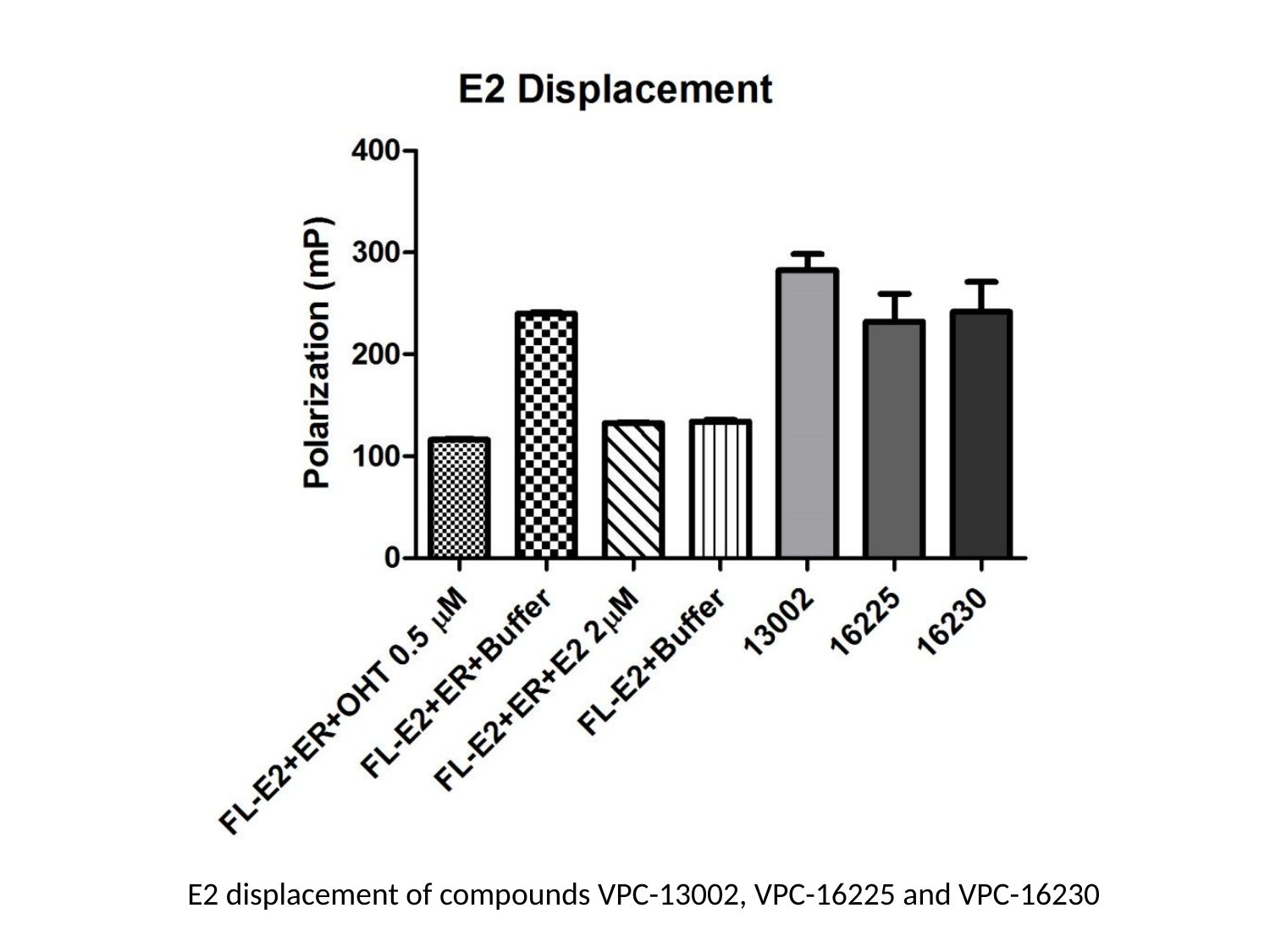

E2 displacement of compounds VPC-13002, VPC-16225 and VPC-16230

Supplement: Additional file 3: Figure S1. — E2 displacement of VPC-13002, VPC-16225 and VPC-16230. The lead compounds were tested at 20 μM for E2 displacement in fluorescence polarization assay. The compounds did not displace Fl-E2. OHT (0.5 μM) and E2 (2 μM) were used as positive controls. [file 13058_2015_529_MOESM3_ESM.pptx]

## Slide 1
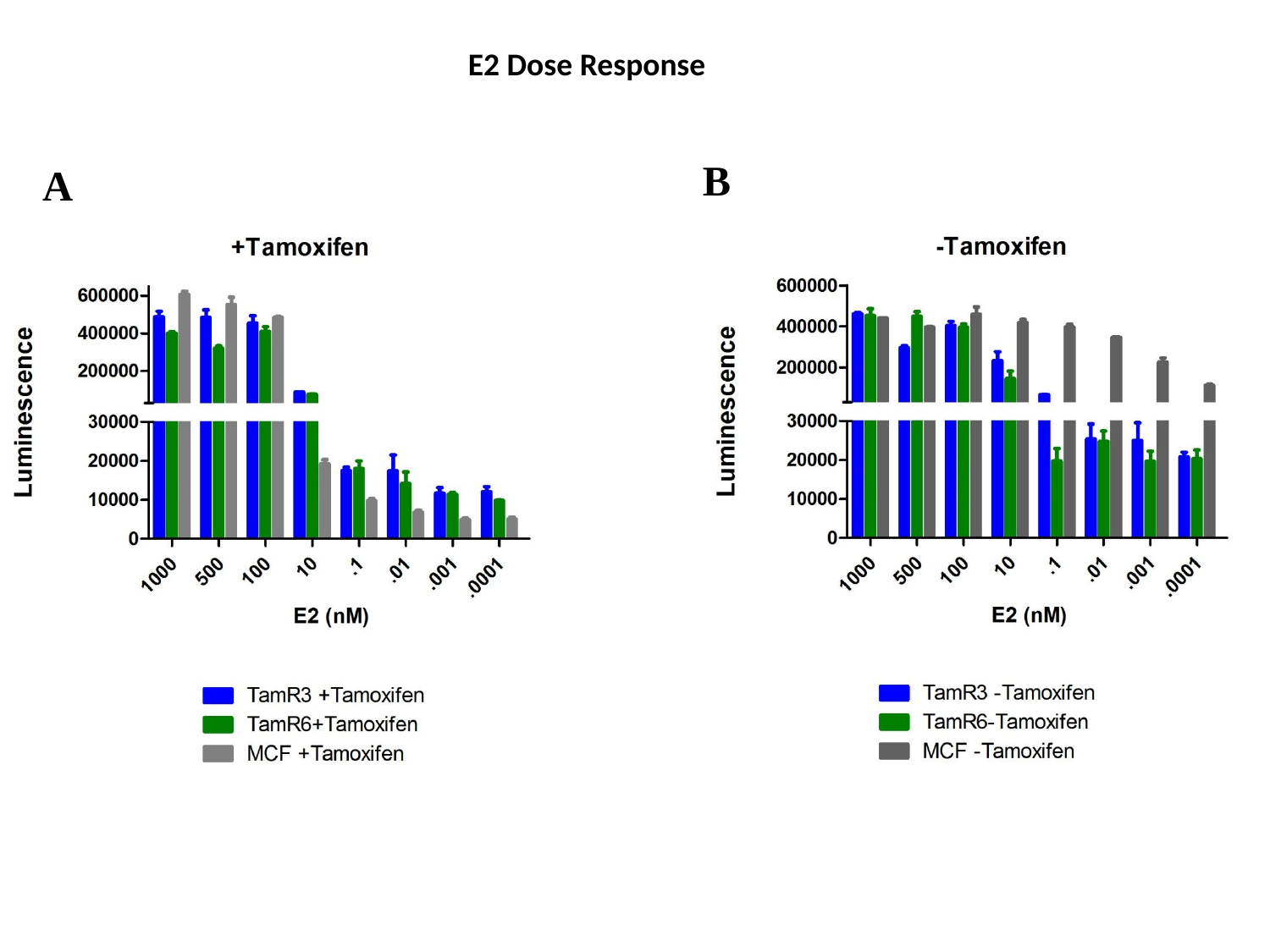

E2 Dose Response
B
A

Supplement: Additional file 4: Figure S2. — E2 dose–response profile of MCF7, TamR3 and TamR6. Dose response to E2 was measured in the three cell lines by luciferase reporter assay using the E2-responsive 3X ERE TATA luc plasmid. (A) In the presence of 1 μM tamoxifen, both MCF7 and TamR cell lines show a dose-dependent response to E2; however, in MCF7, the effect is diminished at lower concentrations of E2. (B) In the absence of 1 μM tamoxifen, MCF7 shows a dose-dependent behaviour with a significantly higher luminescent signal, whereas in TamR cell lines the response is similar to that in the presence of 1 μM tamoxifen. [file 13058_2015_529_MOESM4_ESM.pptx]

## Slide 1
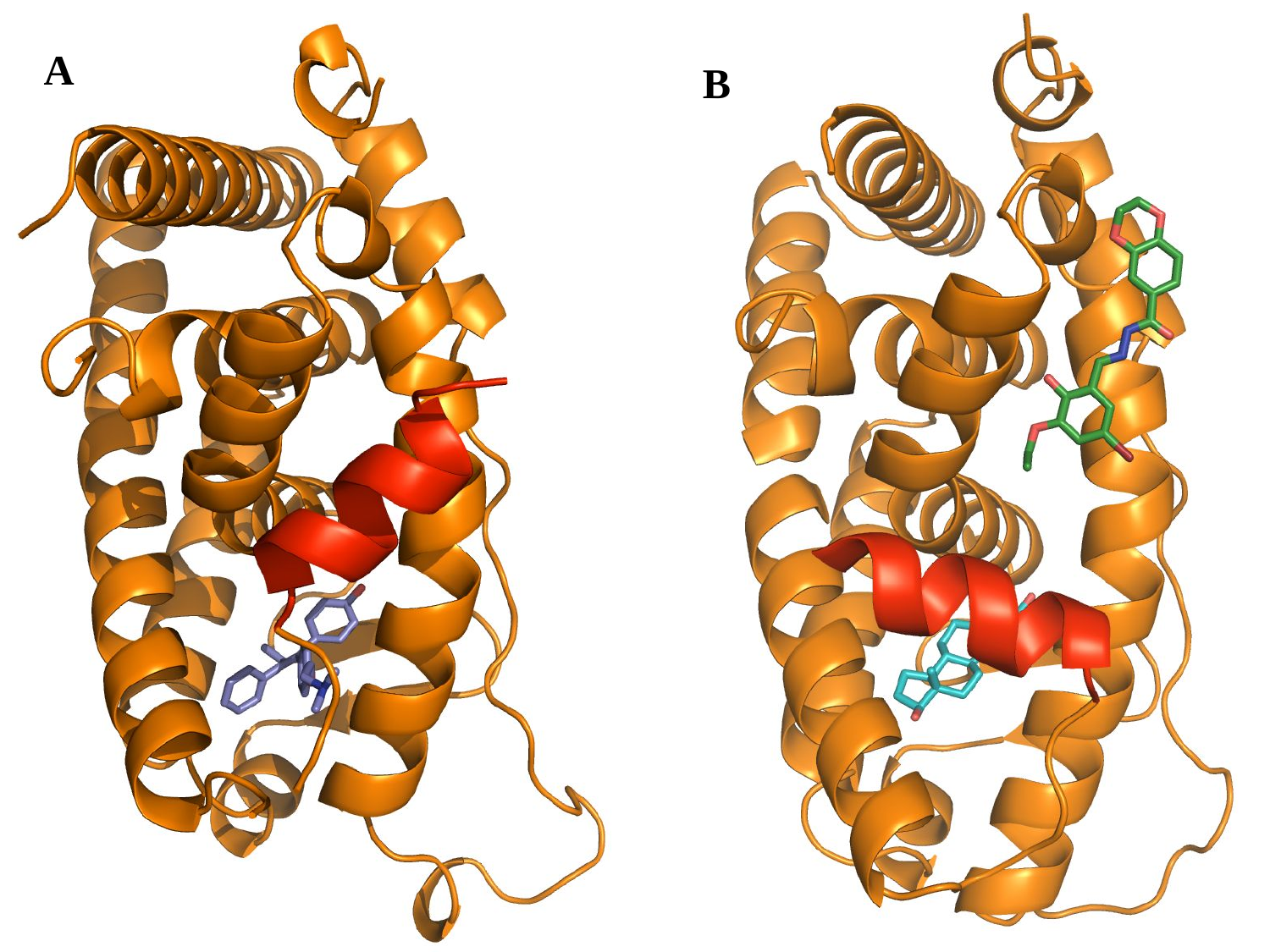

A
B

Supplement: Additional file 5: Figure S3. — Antagonist and agonist models of estrogen receptor-α. (A) Binding of tamoxifen (shown in purple) to ER-α leads to an antagonist conformation, which results in repositioning of αα-helix 12 (show in red) and prevents AF2 formation. (B) Binding of E2 (shown in cyan) causes the movement of α-helix 12 such that it opens the AF2 pocket. In this scenario, AF2 inhibitor (shown in green) directly prevents interaction between coactivators and the receptor. [file 13058_2015_529_MOESM5_ESM.pptx]
